# Ginsenoside Rg1 as a Multifunctional Therapeutic Agent: Pharmacological Properties, Molecular Mechanisms and Clinical Perspectives in Complementary Medicine

**DOI:** 10.1002/fsn3.71486

**Published:** 2026-02-03

**Authors:** Hernán Cortés, Enrique Lima, Lorena Duarte‐Peña, Sheila I. Peña‐Corona, Zainab M. Almarhoon, Rajesh Kaverikana, Shivaprasad Shetty Mangalpady, Vinayaka Babu Shet, Nikshitha Manjeshwar, Javad Sharifi‐Rad, Jen‐Tsung Chen, Gerardo Leyva‐Gómez, William N. Setzer, Daniela Calina

**Affiliations:** ^1^ Laboratorio de Medicina Genómica, Departamento de Genómica Instituto Nacional de Rehabilitación Luis Guillermo Ibarra Ibarra Ciudad de México; ^2^ Laboratorio de Fisicoquímica y Reactividad de Superficies (LaFReS), Instituto de Investigaciones en Materiales Universidad Nacional Autónoma de México Ciudad de México Mexico; ^3^ Departamento de Farmacia, Facultad de Química Universidad Nacional Autónoma de México Ciudad de México Mexico; ^4^ Department of Chemistry, College of Science King Saud University Riyadh Saudi Arabia; ^5^ Nitte (Deemed to be University), NGSM Institute of Pharmaceuticals, Department of Pharmacology Mangaluru India; ^6^ Nitte (Deemed to be University), NMAM Institute of Technology (NMAMIT), Department of Chemistry Nitte India; ^7^ Nitte (Deemed to be University), NMAM Institute of Technology (NMAMIT), Department of Biotechnology Engineering Nitte India; ^8^ Universidad Espíritu Santo Samborondón Ecuador; ^9^ Department of Medicine, College of Medicine Korea University Seoul Republic of Korea; ^10^ Centro de Estudios Tecnológicos y Universitarios del Golfo Veracruz Mexico; ^11^ Department of Life Sciences National University of Kaohsiung Kaohsiung Taiwan; ^12^ Aromatic Plant Research Center Lehi Utah USA; ^13^ Department of Chemistry University of Alabama in Huntsville Huntsville Alabama USA; ^14^ Department of Clinical Pharmacy University of Medicine and Pharmacy of Craiova Craiova Romania

**Keywords:** aging, antioxidant activity, clinical applications, ginseng, ginsenoside Rg1, molecular mechanisms, neuroprotection, pharmacological properties

## Abstract

Ginsenoside Rg1 (GRg1), a major bioactive component of *Panax ginseng* , exhibits potent antioxidant, anti‐inflammatory, and neuroprotective properties, positioning it as a promising therapeutic agent in neurodegenerative and metabolic disorders. This review critically examines the current literature on GRg1, emphasizing its molecular mechanisms, pharmacological pathways, and clinical translation in complementary medicine. GRg1 demonstrates protective effects in conditions such as Alzheimer's disease (AD), Parkinson's disease (PD), ischemic stroke, cardiovascular dysfunction, diabetes, and aging, acting primarily through the nuclear factor kappa B (NF‐κB), mitogen‐activated protein kinase (MAPK), Wnt/β‐catenin, and peroxisome proliferator‐activated receptor gamma/heme oxygenase‐1 (PPARγ/HO‐1) signaling pathways. Evidence from in vitro, in vivo, and clinical studies indicates that GRg1 enhances cellular resilience, reduces oxidative damage, and regulates apoptosis. Despite its broad therapeutic potential, low bioavailability remains a major limitation, warranting the development of advanced delivery systems such as nanoparticles and liposomes. Overall, this review provides a comprehensive assessment of GRg1's pharmacological actions and highlights its growing relevance as a multifunctional therapeutic agent in complementary and integrative medicine.

## Graphical abstract

Ginsenoside Rg1 (Rg1) from *Panax ginseng* shows multifunctional health effects. Although oral bioavailability is low and blood–brain barrier (BBB) penetration is limited, delivery systems such as liposomes and nanoparticles may improve exposure. Rg1 reduces inflammation/oxidative stress by inhibiting nuclear factor‐κB (NF‐κB) signaling and reactive oxygen species (ROS), supporting neuroprotection in Alzheimer's disease (AD), Parkinson's disease (PD), and stroke, alongside cardioprotective, hepatoprotective (non‐alcoholic fatty liver disease, NAFLD), and metabolic benefits.

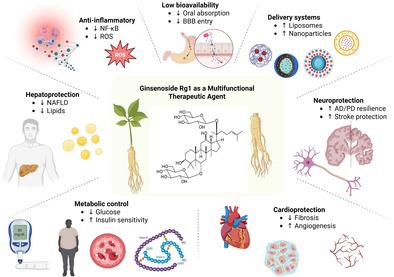

**Abbreviations**
AD: Alzheimer's disease
Akt: Protein Kinase B
AMP: Adenosine Monophosphate
AMPK: AMP‐activated Protein Kinase
APP/Aβ: amyloid precursor protein/amyloid‐β
AUC: area under the curve
BBB: blood–brain barrier
[Ca^2+^]_i_
: intracellular calcium concentration
CaN: Calcineurin
CaSR: calcium‐sensing receptor
Cl: clearance
*C*_max_
: maximum Concentration
CNS: central nervous system
CYP: Cytochrome P450
CYP‐7A: Cholesterol 7 alpha‐hydroxylase
DMT1: type 1 diabetes mellitus
DMT2: type 2 diabetes mellitus
EMT: epithelial‐mesenchymal transition
FoxO1: Forkhead Box Protein O1
FSH: follicle‐stimulating hormone
G6Pase: glucose‐6‐phosphatase
GLUT4: glucose transporter Type 4
GRg1: ginsenoside Rg1
GSK‐3β: glycogen synthase kinase 3 beta
Haspin: haploid germ cell‐specific nuclear protein kinase
HD: Huntington's disease
HO‐1: heme oxygenase 1
HSC: hepatic stellate cell
IC_50_
: half maximal inhibitory concentration
IL‐1β: interleukin‐1 beta
LDL: low‐density lipoprotein
LH: luteinizing hormone
LPS: lipopolysaccharide
MAPKs: mitogen‐activated protein kinases
MPTP: 1‐Methyl‐4‐phenyl‐1,2,3,6‐tetrahydropyridine
mRNA: messenger ribonucleic acid
MRT: mean residence time
NF‐κB: nuclear factor kappa‐light‐chain‐enhancer of activated B cells
nNOS: neuronal nitric oxide synthase
NO: nitric oxide
OGD: oxygen–glucose deprivation
PC12: pheochromocytoma cell line
PD: Parkinson's disease
PDGF: platelet‐derived growth factor
PEPCK: phosphoenolpyruvate carboxykinase
PINK: PTEN‐induced putative kinase
PPARγ: Peroxisome proliferator‐activated receptor gamma
PPD: Protopanaxadiol
SAMP8: senescence‐accelerated mouse prone 8
SMAD: SMA and MAD‐related protein (a family of proteins involved in signal transduction and transcriptional regulation)
Smad7: SMAD family member 7
SNpc: substantia nigra pars compacta
SREBP‐1C: sterol regulatory element‐binding protein 1C
T_1/2_
: Half‐Life
TGF‐β1: transforming growth factor beta 1
TLR: toll‐like receptor
*T*_max_
: time to maximum concentration
TNF‐α: tumor necrosis factor alpha
*V*_d_
: volume of distribution
VEGF: vascular endothelial growth factor

## 1 Introduction

**FIGURE 1:**
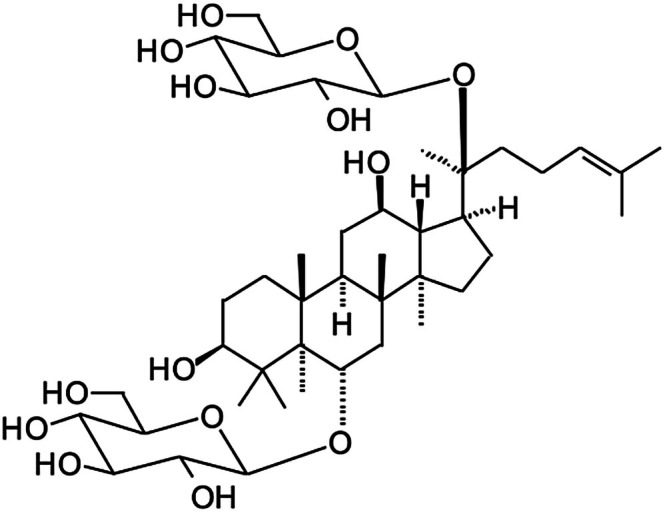
The chemical structure of ginsenoside Rg1.

Chinese ginseng ( *Panax ginseng* C.A. Meyer; family: Araliaceae) is an important medicinal herb widely consumed in Chinese medicine, alone or in combination with others (Leung and Wong 2010). Botanically, *P. ginseng* is native to the cool temperate regions of Northeast China, Korea, and parts of Russia, and thrives in shaded, well‐drained, humus‐rich soils at high altitudes. It is traditionally cultivated under forest or artificial shade conditions to mimic its natural habitat (Liu et al. 2022). The cultivation environment, particularly soil type, climate, and plant age, significantly influences the ginsenoside content and ratio, leading to variability in pharmacological potency. China and South Korea are the leading producers of *P. ginseng* , with increasing cultivation efforts in Canada and Eastern Europe to meet growing global demand (Yuan et al. 2024). Ginseng is known for its beneficial effects on blood pressure, the immune system, and metabolic activities. Ginsenosides are the prime pharmacologically active compounds responsible for the medicinal properties of ginseng, which are also found in many other *Panax* species, including *P. vietnamensis* and *P. notoginseng*. Scientists have identified more than 30 ginsenosides, named “Rx,” where “R” refers to “root” and “x” defines the compound's polarity. The compounds are classified into 2 groups: PPD (20(S)‐protopanaxadiol), which includes Rb1, Rb2, Rc, Rd, Rg3, Rh2, Rs1, among others, and the PPT (20(S)‐protopanaxatriol), which contains Re, Rf, Rg1, Rg2, and Rh1. The presence of the C‐6 carboxyl group in PPDs makes them different from PPTs. In the current review, ginsenoside Rg1 (GRg1) (C_42_H_72_O_14_) (Figure 1) is the compound of interest.

The current review focuses on GRg1, a PPT‐type ginsenoside with the molecular formula C_42_H_72_O_14_ (Figure 1). GRg1 has gained significant attention due to its multifaceted therapeutic potential, particularly in neurodegenerative disorders, ischemic stroke, metabolic regulation, inflammation, aging, and cardiovascular diseases (Abdelilah 2025). GRg1 exerts its effects by modulating various signaling pathways, demonstrating antioxidant, anti‐inflammatory, and neuroprotective properties (Xiong Yang et al. 2021). Additionally, GRg1 is primarily eliminated via a hepatobiliary pathway and has been shown to protect nerve cells by reducing apoptosis, highlighting its potential as a neuroprotective agent (Junxiong et al. 2010). This review provides a comprehensive analysis of GRg1's pharmacological properties, particularly emphasizing its therapeutic applications across multiple disease models. The article uniquely integrates findings from in vitro, in vivo, and clinical studies, offering a detailed exploration of GRg1's mechanisms of action. This review not only consolidates current knowledge but also identifies gaps in existing research, paving the way for future studies on GRg1's therapeutic potential in modern medicine.

## 2 Methodology

Information about GRg1 was searched in electronic databases, such as PubMed/MedLine, Google Scholar, Web of Science, and Scopus. Key search terms and MeSH terms included “ginsenoside Rg1,” “bioavailability,” “pharmacokinetics,” “mechanism of action,” “neurodegenerative diseases,” “hepatoprotective,” “anti‐diabetes,” “anti‐aging,” “anti‐inflammatory,” “anti‐cancer,” “clinical applications,” and “mechanisms of action.” The search was restricted to articles in English published in peer‐reviewed journals. Articles were included if they considered the GRg1 therapeutic effects and mechanisms. The inclusion criteria encompassed in vitro and in vivo studies, both clinical and non‐clinical, with no restriction on the publication date to ensure a comprehensive understanding of the field. Only accessible full texts were considered. Excluded from the review were articles not directly related to GRg1, opinions, editorials, and review articles. The article selection process began with a preliminary screening of titles and abstracts for relevance to the study objectives. A full‐text review of the qualified articles was needed for a more comprehensive assessment. Data extraction was focused on pharmacological properties, the experimental model in vitro/in vivo, administration and dosage, key findings, effects, and GRg1 action mechanisms.

## 3 Bioavailability and Pharmacokinetics of GRg1

The oral bioavailability of GRg1 is very low, which can be attributed to low permeability, absorption, first‐pass metabolism, and hepatic clearance (Han and Fang 2006; Laccourreye and Maisonneuve 2019). Pharmacokinetic parameters of GRg1 were studied in rats using a total *Panax* notoginsenoside by oral and IV route (Li et al. 2007). The authors reported that the absolute bioavailability of GRg1 after oral administration of total *Panax* notoginsenoside was 6.06% (*T*_max_ of 0.75 ± 0.00 h, *C*_max_ of 6.42 ± 1.74 mg/L; *T*_1/2_ of 5.01 ± 2.09 h). On IV administration, *V*_d_ was reported to be 15.60 ± 12.06 L/kg, *T*_1/2_ 4.03 ± 2.75 h, and Cl was reported to be 1.91 ± 0.33 L/h/kg. After oral administration, GRg1 was detected in blood in 15 min, and *C*_max_ was achieved within 1 h of the administration. The authors also estimated the GRg1 concentration in different organs after IV injections and reported the maximum concentration in the liver after 5 min. Kidneys, heart, lungs, spleen, and pancreas were also reported to have GRg1 in high concentration, but brain permeation was reported to be minimal. The authors concluded that the low BBB permeability could be the reason for the low concentration of GRg1 in the brain (Feng, Wang, et al. 2010; Won et al. 2019). Metabolic pathways of GRg1 were studied in rats. The authors reported that it is quickly metabolized in vivo by multiple deglycosylations, leading to the formation of protopanaxatriol (Won et al. 2019). In another study, bioavailability in rats of GRg1 and metabolites was estimated after oral and IV dose administration. The results suggested that after oral administration, metabolites of GRg1 were detected in plasma in abundance compared to the parent compound with a better pharmacokinetic parameter than GRg1 (*T*_max_: GRg1 = 0.92, RH1 = 3.64, F1 = 5.17, and protopanaxatriol (Ppt) = 7.30 h; MRT were GRg1 = 2.68, RH1 = 5.06, F1 = 6.65, and Ppt = 5.33 h, respectively; AUC (_0‐t_), were GRg1 = 2363.5, RH1 = 4185.5, F1 = 3774.3, and Ppt = 396.2 ng/mL/h, respectively). GRg1 had a better plasma concentration following IV administration than its metabolites (AUC_0‐tS_ were GRg1 = 1454.7, RH1 = 597.5, and F1 = 805.6 ng/mL/h, respectively). The metabolites, however, were detectable for longer compared with the parent compound (T_1/2betaS_ were GRg1 = 3.12, 5.87, and RH1 = 6.87 h, respectively; MRTs were 1.92, 5.99, and 7.13 h, respectively) (Feng, Hu, and Yu 2010). Another study reported that the relative bioavailability of GRg1 was 2.5% compared with the IV dose (Tan et al. 2013). Han & Fang studied the reason for the low oral bioavailability using an in vitro cell line model and an in vivo rat model (Han and Fang 2006). The authors concluded that the elimination of GRg1 in the stomach, large intestine, and liver contributes to low bioavailability, and low permeability is the primary factor resulting in decreased absorption. To address these limitations, several strategies have been proposed in recent literature. Nanocarrier‐based systems, such as liposomes, solid lipid nanoparticles, and polymeric micelles, have demonstrated the ability to enhance solubility and intestinal permeability of poorly absorbed compounds, including ginsenosides. Additionally, encapsulation into microspheres or co‐delivery with permeability enhancers and enzyme inhibitors may reduce first‐pass metabolism. Prodrug approaches targeting intestinal esterases are also under investigation to improve Rg1's bioactivation. Moreover, modulation of gut microbiota has shown promise in enhancing the transformation of Rg1 into more absorbable forms such as Rh1 and protopanaxatriol. Potential interactions between GRg1 and conventional drugs should be taken into account in its clinical application. GRg1 and other ginseng components may influence drug metabolism by affecting cytochrome P450 activity, intestinal absorption, or plasma protein binding. Some studies have reported reduced plasma concentrations of warfarin and enhanced aspirin bioavailability after ginseng intake, indicating possible pharmacokinetic and pharmacodynamic interactions. Therefore, the combined use of GRg1 with anticoagulants, antidiabetic agents, or central nervous system drugs requires caution (Sun, et al. 2022). More studies are needed to confirm these effects and ensure safe therapeutic use. These approaches, individually or in combination, represent promising avenues to overcome Rg1's low oral bioavailability and warrant further investigation in preclinical and clinical settings.

## 4 Pharmacological Properties

### 4.1 Neuroprotective Effects

#### 4.1.1 Neurodegenerative Diseases

##### 4.1.1.1 A Brief Overview of Key Molecular Mechanisms

**FIGURE 2:**
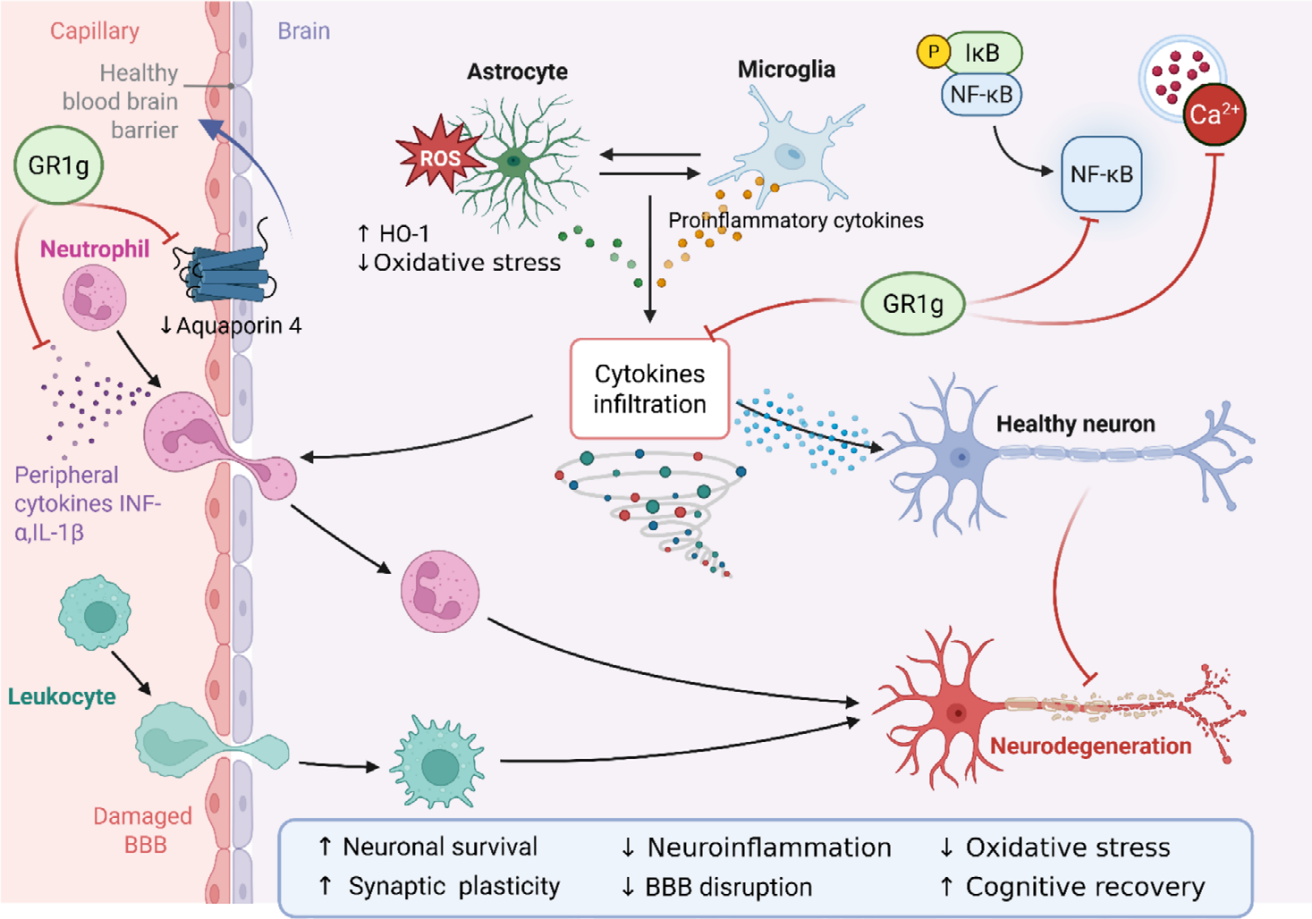
Neuroprotective and anti‐ischemic mechanisms of GRg1 in neurodegenerative diseases. GRg1 protects neurons by modulating inflammation, oxidative stress, and ischemic injury pathways. It reinforces BBB integrity by reducing Aquaporin‐4 expression and cytokine infiltration, inhibits NF‐κB signaling in microglia, enhances HO‐1 antioxidant activity in astrocytes, and limits Ca^2+^‐induced neuronal damage. These actions collectively decrease neuroinflammation and oxidative stress while supporting neuronal survival, synaptic plasticity, and cognitive recovery. Abbreviations and symbols: → activation or promotion; ⊣ inhibition or suppression; red arrows: Pathological effects; blue/green arrows: GRg1‐mediated protective actions; ↑, Increase; ↓, Decrease; BBB, Blood–brain barrier; Ca^2+^, Calcium ion; GRg1, Ginsenoside Rg1; HO‐1, Heme oxygenase‐1; IL‐1β, Interleukin‐1 beta; IκB, Inhibitor of kappa B; NF‐κB, Nuclear factor kappa B; P, Phosphorylated; ROS, Reactive oxygen species; TNF‐α, Tumor necrosis factor‐alpha.

**FIGURE 3:**
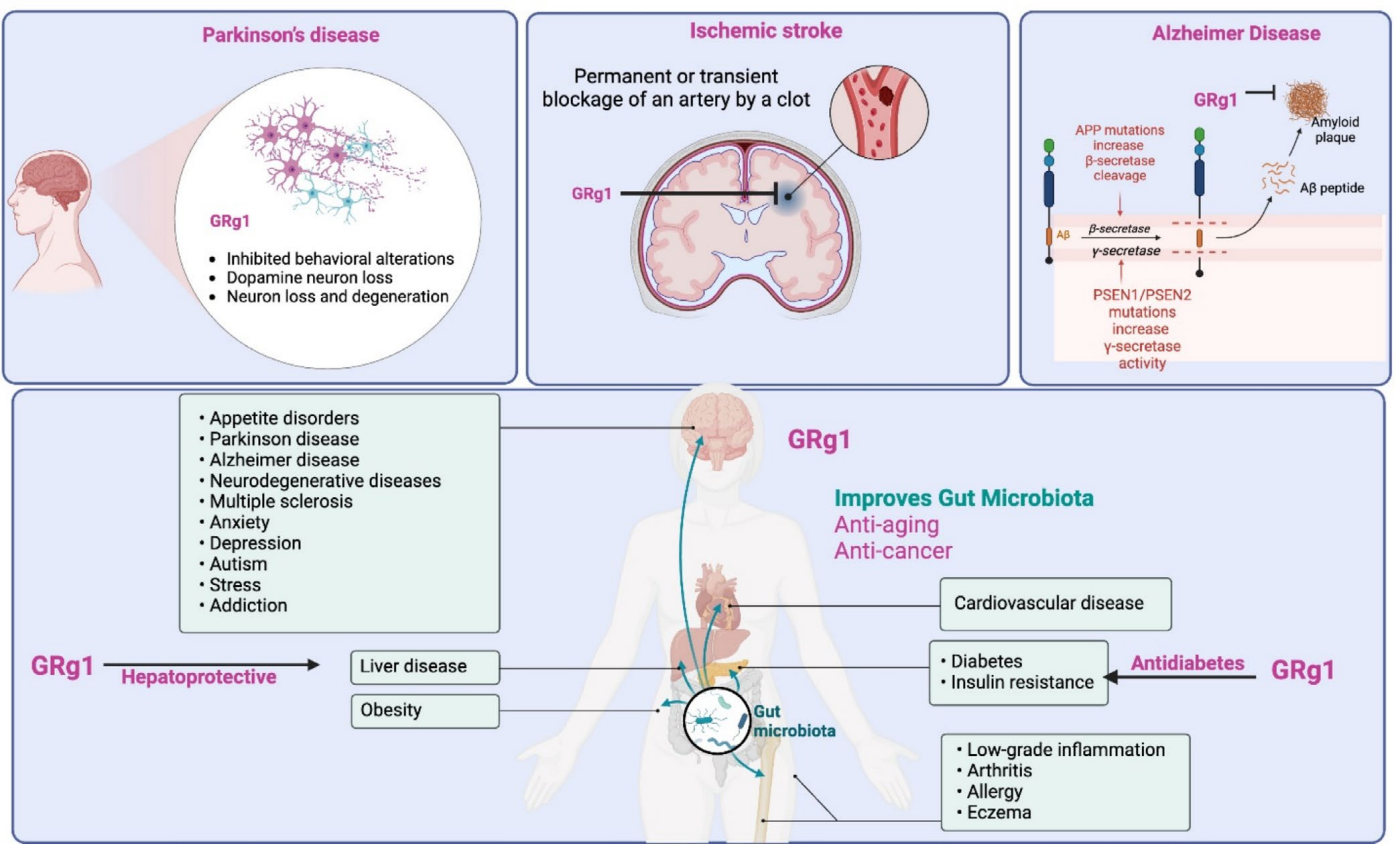
Systemic and disease‐specific therapeutic actions of GRg1. GRg1 exhibits broad therapeutic effects across multiple organ systems and disease models. In the nervous system, GRg1 mitigates dopaminergic neuron loss in Parkinson's disease, reduces amyloid plaque formation in Alzheimer's disease by inhibiting β‐ and γ‐secretase activity, and protects against ischemic stroke‐induced neuronal injury. Beyond the brain, GRg1 demonstrates hepatoprotective, antidiabetic, and anti‐obesity effects, primarily through modulation of oxidative stress, lipid metabolism, and insulin sensitivity. Moreover, GRg1 supports gut microbiota balance, contributing to anti‐aging, anti‐cancer, and anti‐inflammatory benefits, thereby highlighting its multifunctional potential in both neurological and metabolic disorders. Aβ, Amyloid‐beta; APP, Amyloid precursor protein; BBB, Blood–brain barrier; GRg1, Ginsenoside Rg1; Ppt, Protopanaxatriol; PSEN1/PSEN2, Presenilin 1/Presenilin 2.

According to traditional Chinese medicine, ginseng can calm the mind and promote wisdom. GRg1 is known to have antioxidant, anti‐inflammatory, and neuroprotective actions, and it has been thoroughly investigated for its beneficial effects on various central nervous system (CNS) disorders, with a particular emphasis on depression and dementia (Yang et al. 2023) (Figure 2, Table 1, Figure 3).

**TABLE 1:** Effects and pharmacological mechanisms of action of ginsenoside Rg1.

Pharmacological properties	Experimental model in vitro/in vivo	Administration and dosage IC_50_ for in vitro/dose for in vivo	Effects and mechanisms	References
Anti‐cancer (Inhibiting EMT)	HepG2 liver cancer cells	Not available	Inhibited the expression of the mesenchymal phenotype marker vimentin by inhibiting TGF‐*β*1 recovered the expression of E‐cadherin	Yu et al. (2015)
Alzheimer's disease	5XFAD mouse model	10 mg/kg/d	GRg1 induces PINK‐Parkin‐mediated mitophagy and ameliorates memory deficits in 5XFAD	Wang et al. (2023)
Cardioprotective	Rat model	Not available	The activation of the CaN pathway and the increase of [Ca^2+^]_i_ mediated by CaSR are involved in cardiac hypertrophy and fibrosis.	Lu et al. (2021)
Hepatoprotective	Mouse model	40 mg/kg in vivo	GRg1 inhibits HSC activation by epigenetically modulating Smad7 expression	Zhang et al. (2023)
Antiaging	Human (in vivo)	5 mg	GRg1 supplementation effectively eliminates senescent cells in exercising human skeletal muscle and improves high‐intensity endurance performance	Wu et al. (2019)
Anti‐inflammatory	Rats	150 mg/kg/day	Prevent dehydroepiandrosterone‐induced polycystic ovarian syndrome via anti‐inflammatory and antioxidant activities	Choi et al. (2020)
Anti‐cancer	HeLa (uterus adenocarcinoma), MDA‐MB‐231, MCF‐7 (breast cancer cell lines), H226B, H226Br, H1299, A549 (lung cancer cell lines), SW480, HCT116 (colon cancer cell lines)	10 μM dose	GRg1 reduces the level of Aurora B at the centromere via perturbing Haspin kinase activity and concurrent H3T3ph.	Hong et al. (2022)

*Note:* [Ca^2+^]_i_ intracellular calcium concentration, Aurora B Aurora Kinase B, CaN Calcineurin, CaSR calcium‐sensing receptor, EMT epithelial‐mesenchymal transition, H3T3ph phosphorylation of histone H3 at threonine 3, Haspin Haploid Germ Cell‐Specific Nuclear Protein Kinase, HSC hepatic stellate cell, IC_50_ half maximal inhibitory concentration, PINK PTEN‐induced putative kinase, Smad7 SMAD family member 7 and TGF‐β1 transforming growth factor beta 1.

The molecular mechanisms underlying these effects converge on several key signaling pathways that act as central regulatory nodes in neuroprotection and inflammation control. Among these, the NF‐κB and MAPK cascades serve as principal mediators of Rg1's anti‐inflammatory and anti‐apoptotic effects, suppressing proinflammatory cytokine production and microglial activation in neurodegenerative conditions (Wang et al. 2025). The Wnt/GSK‐3β/β‐catenin axis is repeatedly implicated as a critical pathway promoting neuronal survival, synaptic plasticity, and cognitive improvement, while the PPARγ/HO‐1 pathway plays a key antioxidative and cytoprotective role by modulating oxidative stress and apoptotic signaling. Collectively, these interconnected pathways form the mechanistic foundation for Rg1's diverse pharmacological actions across multiple systems. Alzheimer's disease (AD) is a result of a neurodegenerative process, which is associated with beta‐amyloid deposition and neurofibrillary tangles formation (Wang et al. 2025). Clinically, this results in altered memory, attention, cognition, and judgment, adversely affecting individuals’ quality of life. Scientists are still in search of any satisfactory preventive or therapeutic intervention for AD. In this regard, the action of GRg1 as a memory enhancer and neuroprotective has gained the contemplation of scientists (Guo et al. 2021; Ong et al. 2015).

##### 4.1.1.2 GRg1 in AD

In transgenic AD mice overexpressing amyloid precursor protein/amyloid‐β (APP/Aβ), GRg1 treatment markedly reduced cerebral Aβ accumulation and ameliorated neuronal pathology, resulting in preserved memory and improved spatial learning (Fang et al. 2012), mechanistically, GRg1 hastens the activation, nuclear transcription, and translocation of NF‐κB, thereby enhancing the NF‐κB binding with corresponding sites at promoter regions of DNA, which is responsible for a β‐secretase gene. In turn, GRg1 reduces the production of Aβ by suppressing the transcription and translation of this gene. Recent studies have identified Aβ as an antimicrobial peptide with its share in the innate immune response (Kowalski and Mulak 2019). Gut microbiota have been identified as an essential player in the development and progression of AD. Bacterial metabolites may moderate microglia development, Aβ clearance, and the protection of neurons (Kowalski and Mulak 2019; Mosher and Wyss‐Coray 2014). A study on rats inoculated with *Escherichia coli* resulted in Aβ deposition in the gut and neurons in the brain, producing curly amyloid fibrils, and glial cell and astrocyte proliferation was also reported to be enhanced (Chen et al. 2016). Changes in gut microbial fabric and alterations induced by broad spectral antimicrobial therapy for a prolonged period were shown to reduce the Aβ plaque deposition and abnormal increase in glial cells (Minter et al. 2016). Studies conducted on tree shrews concluded that GRg1 modifies the gut microbiota in addition to inhibiting pro‐apoptotic protein expression and neuroprotective action (Guo et al. 2021). Another survey of tree shrews showed that GRg1, by modulating Wnt/GSK‐3β/β‐catenin pathway, attenuates damage due to oxidative stress, relieves neuroinflammation, and exhibits neuroprotective action, leading to improvement in cognitive function in AD (Yang et al. 2022). Scientists have identified different mechanisms by which GRg1 may alter the disease initiation and progression in AD. Several mRNAs have been identified, which can affect the disease onset and progression of AD. One study claims GRg1 suppresses apoptosis by controlling the miR‐873‐5p in AD (Shi et al. 2018). Dysregulation of heme oxygenase 1 has been recognized to play a role in cognitive impairment in AD. Reports suggest that the inhibitors of heme oxygenase 1 provide neuroprotective benefits in AD (Gupta et al. 2014; Shi et al. 2018; Sung et al. 2016) and revealed that GRg1 could control the expression of heme oxygenase 1 by enhancing the miR‐873‐5p levels. Thus, GRg1 provokes beneficial effects in AD. Another study, which utilized NG108‐15 cell lines, concluded that GRg1 exhibits its anti‐AD action by inhibiting TLR3 and TLR4 signaling pathways and reducing the proinflammatory factors instigated by Aβ25‐35 in the cell lines (Zhao et al. 2014).

##### 4.1.1.3 GRg1 in Huntington's Disease

Huntington's disease (HD) is a hereditary neurodegenerative disorder caused by mutations in the *huntingtin* gene, leading to striatal neuron loss and motor dysfunction (Walter et al. 2016; Zuccato et al. 2010). Clinical manifestations include motor function abnormalities, cognitive impairment, neuropsychiatric manifestations, and motor dysfunction (Snowden 2017). Pathologically, neuron loss in the striatum is the major feature of HD, leading to progressive atrophy of the striatum, involuntary muscle movements, and coordination defects (Dargaei et al. 2018; Sepers et al. 2018). GRg1 was also studied for its protective action in HD (Yang et al. 2021). The study involved 3‐NP to induce the disease in the mouse model. The results show that pre‐treatment of animals with GRg1 attenuated the 3‐NP‐induced behavioral alterations and weight loss. Pre‐treatment also suppressed 3‐NP‐caused nerve loss and structural and morphological injury in the brain's striatum. Furthermore, the 3‐NP‐caused apoptosis has been reported, and activation of microglia and inflammatory mediators in the striatum were also suppressed. The study attributes the disease development prevention mechanism to the suppression of 3‐NP–mediated activation of MAPKs and NF‐κB signaling pathway by the GRg1 in the striatum.

##### 4.1.1.4 GRg1 in Parkinson's Disease

Parkinson's disease (PD) is characterized by the progressive degradation of dopaminergic neurons, especially in the substantia nigra pars compacta (SNpc), and the existence of intraneuronal proteinaceous substance, Lewy bodies, and Lewy neuritis (Heng et al. 2016). Oral administration of GRg1 in an MPTP‐treated mouse model for PD effectively inhibited mortality, behavioral alterations, and dopamine neuron loss and inhibited ultrastructure abnormalities in the SNpc. MPTP‐induced neuroinflammation was also reported to be attenuated. The authors attribute the protective effect of GRg1 to its anti‐neuroinflammatory actions. They further reported that GRg1 controlled the MPTP‐mediated reactive astrocytes and microglia and inhibited cytokines like TNF‐α and IL‐1β in the SNpc (Heng et al. 2016). In another study, an in vitro model for PD was carried out using the PC12 cell line; adding MPP+ to the culture medium caused apoptosis, whereas GRg1 could effectively inhibit this action. The authors also demonstrated that this protective action of GRg1 is due to the activation of the Wnt/β‐catenin pathway, and adding a specific blocker to the cell culture resulted in the blockade of GRg1's protective action (Zhou et al. 2016). In the lipopolysaccharide‐induced brain damage model (microglia activation and dopaminergic neuronal degeneration in the substantia nigra of rats), treatment with GRg1 delivered good protection against the LPS‐induced brain damage. A specific glucocorticoid receptor antagonist RU486 could abolish this protective action of GRg1 to a significant extent, and the author suggests that the modulation of GR signaling pathways is one of the essential mechanisms of the anti‐PD action of GRg1 (Sun, Ren, et al. 2016).

#### 4.1.2 Stroke

Ischemic stroke is the result of the persistent or brief curtailment of blood flow and is one of the significant causes of morbidity and mortality around the world (Gupta and Wagh 2023; Naghavi et al. 2024), and the major neurological damage is believed to be due to ischemia–reperfusion‐related injury (Sharifi‐Rad et al. 2022). A generally accepted theory is that apoptosis is the molecular mechanism associated with reperfusion injury; the most susceptible regions for reperfusion injury‐induced apoptosis in the brain include pyramidal neurons in the hippocampal region (Einenkel and Salameh 2024). A study on rats was performed to evaluate the involvement of PPARγ/HO‐1 signaling in reperfusion injury after ischemic stroke. The study also assessed the effect of GRg1 on this. The authors reported that the PPARγ/HO‐1 signaling pathway plays an essential role in the apoptosis of nerve cells after an ischemic stroke and following reperfusion injury. They also demonstrated that GRg1, by activating the PPARγ/HO‐1 signaling pathway, provides significant protection against apoptosis and inflammation in the hippocampus, resulting in structural and functional brain damage (Yang et al. 2015). The protective effect of GRg1 against reperfusion injury was studied in hippocampal cell culture. The authors concluded that GRg1 exhibited significant protection against ischemic‐reperfusion injury, which was brought about by inhibiting the calcium over‐influx to the neurons and by reducing nNOS (neuronal nitric oxide synthase) activity following OGD (oxygen–glucose deprivation). The mechanism involved in this case seems to be the inhibition of calcium influx and decreased nNOS activity (He et al. 2014; Sun et al. 2014). A study on a rat reperfusion injury model was carried out to estimate GRg1's ability to deliver neuroprotection. The reports indicated that GRg1 could provide significant protection, improve neurological functions in ischemic reperfusion injury, and decrease BBB disruption following focal cerebral ischemia. The mechanism of this protective action of GRg1 was noted to be through the downregulation of aquaporin‐4 expression (Zhou et al. 2014).

### 4.2 Cardioprotective

Bioactive compounds have shown promising cardioprotective effects, offering potential therapeutic strategies for managing cardiovascular diseases. GRg1 protects cardiovascular system functions during the ailment. Rg1 has reported significant physiological and clinical effects in rats for attenuating left ventricle myocardial fibrosis and maintaining cardiac function by enhancing angiogenesis and myocardial structure (Li, Pan, et al. 2018). During the animal study experiments, vascular remodeling was inhibited by Rg1 to perform cardio protection along with slight resistance and large conductance arteries (Chen et al. 2012). Doxorubicin‐induced cardiotoxicity was prevented by Rg1 through anti‐apoptosis in mice, leading to the potential clinical exploration (Zhu et al. 2017). In vivo and in vitro studies revealed pharmacological efficacies to antidiabetic, anti‐cancer, neuroprotective, and cardiovascular activities by Rg1 (Mohanan et al. 2018). GRg1 was administered to the carotid artery injury model, and tissues damaged due to neointimal thickening and aortic injury were improved. It was possibly by oxidative stress reduction to inhibit neointimal hyperplasia (Gao et al. 2018). Platelet aggregation and activation induced by thromboxane analog U46619, collagen, ADP, and thrombin were inhibited by GRg1. The suppression was achieved on extracellular signal‐regulated kinase (Sun, Ren, et al. 2016). GRg1 is favorable in protecting ultra‐structure integrity, hypertension induced due to cardio‐fibrosis, and impairment of hypertensive heart (Chen et al. 2012).

### 4.3 Hepatoprotective

One of the most common liver diseases, nonalcoholic fatty liver disease, occurs in people who do not have a history of alcohol intake (Cioboată et al. 2017). The development and progression of this disease involve complex mechanisms, which include oxidative stress, metabolic impairment, especially in lipid metabolism, inflammatory conditions, and similar pathophysiological features as with metabolic syndrome and type 2 diabetes (Stan et al. 2023). Reports suggest GRg1 protects against non‐alcoholic fatty liver disease by upregulating PPARα (peroxisome proliferator‐activated receptor—alpha). This leads to the stimulation of fatty acid beta‐oxidation and, in turn, promotes free fatty acid metabolism and triglyceride metabolism (Xu et al. 2018). Another study, which further evaluated the mechanical aspects of GRg1 protection against non‐alcoholic fatty liver disease, concluded that GRg1 suppresses inflammation and attenuates liver damage. GRg1 also promoted the expression of different enzymes, including CPT1A, CPT2, and CYP‐7A. It suppressed the expression of SREBP‐1C by enhancing the PPARα expression and modulating the metabolism of lipids, leading to protection against the disease (Hou et al. 2020).

### 4.4 Antidiabetes

Natural compounds have emerged as promising agents for antidiabetic effects due to their ability to modulate glucose metabolism, enhance insulin sensitivity, and reduce oxidative stress, offering a complementary approach to conventional diabetes treatments (Sati et al. 2024). Obesity, driven by excess nutrient deposition, increased white adipose tissue, and elevated cortisol levels, significantly predisposes individuals to type 2 diabetes and other metabolic conditions (Popescu et al. 2022). Many reports suggest GRg1 could be helpful in obesity and type‐2 diabetes (Li, Zhang, et al. 2018). In this regard, an extensive study reported that GRg1 could effectively inhibit the high‐fat diet–induced accumulation of fat in the adipose tissue of mice and inhibit fasting blood glucose and postprandial glucose levels. GRg1 also ameliorates insulin resistance and glucose intolerance in mice. GRg1 activates the AMPK pathway in vitro and in vivo and enhances plasma membrane translocation of GLUT4 in the mouse myoblast cell line C2C12. The mechanism of anti‐obesity and anti‐insulin resistance action of GRg1 can be attributed to the activation of the AMPK pathway (Li, Zhang, et al. 2018). A study conducted on nematode *Caenorhabditis elegans* reported GRg1's ability to suppress the accumulation of lipids and enhanced stress resistance. This was noted to be an effect of a significant reduction in the expression of genes related to fatty acid synthesis and genes responsible for lipid metabolism. With the help of network pharmacology, the authors demonstrated the possible pathway that targets lipid metabolism and increases the expression of anti‐oxidative genes as well as heat shock proteins in *C. elegans*, which can be attributed to resistance to stress (Shi et al. 2023). In a high‐fat diet mouse model, high fasting glucagon concentrations were reported, leading to high plasma glucose levels in fasting animals. Increased plasma glucagon production in fasting animals resulted in deactivated Akt and enhanced glucose production in the liver via FoxO1 activity upregulation. The GRg1, by binding to Akt at a specific site, conserved Akt activity in glucagon‐impaired animals. Akt binding to FoxO1 was also promoted by GRg1, leading to the inactivation of FoxO1 by phosphorylation. It reduces glucose production in the liver by decreasing the transcription of phosphoenolpyruvate carboxykinase (PEPCK) and glucose‐6‐phosphatase (G6Pase) (Liu, Zhang, et al. 2017).

### 4.5 Anti‐Aging

GRg1, a principal bioactive constituent of ginseng, exhibits anti‐aging effects by mitigating oxidative stress and age‐associated functional decline (Thorpe and Baynes 2003). Clinical studies carried out in vivo and in vitro have demonstrated the anti‐aging effect claim of ginseng (Flanagan et al. 2018). GRg1 was injected into mice in an aging model to investigate the effect on the pancreas, revealing the significant suppression of aging. The results indicated that GRg1 possibly overcame the D‐galactose–triggered aging effect on the mouse's pancreas (Thorpe and Baynes 2003). The impact of D‐galactose–induced damage on the pancreas was significantly protected by Rg1 in mice (Dong, Xu, et al. 2017). By improving anti‐aging, GRg1 has reduced interstitial fibrosis, apoptosis, and endoplasmic reticulum stress in the mice (Ding et al. 2020). The anti‐aging effect of GRg1 was explored on the nervous system. Apoptosis of neuronal cells was inhibited and the proliferation of cells was enhanced (Lai et al. 2018). The anti‐aging effect of GRg1 prevailed in bone marrow stromal cells and the hematopoietic microenvironment (Hu et al. 2015). GRg1, administered to animal models, revealed the protection of neural stem cells. Hippocampal progenitor cell proliferation was regulated by GRg1 to exhibit an anti‐aging effect (Zhou et al. 2020).

### 4.6 Anti‐Inflammatory

The body's defense process is characterized by releasing inflammatory mediators and moving lymphocytes, monocytes, and neutrophils from blood to the site of the affected tissue (Iqbal et al. 2024). Oxidative stress and inflammation are closely interconnected processes that contribute to the pathogenesis of various chronic diseases, and natural compounds with antioxidant and anti‐inflammatory properties offer a promising therapeutic approach to counteract these pathological mechanisms. Neuroinflammatory disorders are treated with GRg1. It has exhibited neuroprotective effects through glucocorticoid and estrogen receptors (Gao et al. 2019). GRg1 interacts with signaling pathways to inhibit the activation of MAPK and NF‐κB, triggering proinflammatory chemokines and cytokine production. Septic encephalopathy is treated with GRg1 (Chen et al. 2022). Rg1 was metabolized into 20(S)‐protopanaxatriol in the gut microbiota through ginsenosides F1 and Rh1. NF‐κB activation was inhibited in LPS‐stimulated macrophages (Lee et al. 2015). The anti‐inflammatory action of GRg1 subsidizes mastitis in goats induced by the lipopolysaccharide (Wang et al. 2019). Rg1 is reported in treating rheumatoid arthritis (Feng et al. 2022).

### 4.7 Anticancer Activity of GRg1

Natural compounds have emerged as promising anticancer agents due to their ability to target multiple signaling pathways, induce apoptosis, and inhibit tumor growth with fewer side effects compared to conventional chemotherapy (Azzini et al. 2024; Ceylan et al. 2025; Nandi et al. 2024; Petran et al. 2024). GRg1 has demonstrated promising anti‐cancer effects by preventing cancer cell proliferation through mitotic arrest and inducing apoptosis (Hong et al. 2022). The administration of GRg1 into D‐galactose–induced liver injury mice reduced tumor growth in the liver (Xiao et al. 2018). Cell proliferation of breast cancer cells was inhibited by GRg1 by inducing reactive oxygen species (Chu et al. 2020). Inhibition of epithelial‐mesenchymal transition (EMT) was reported in liver and ovarian cancer (Liu, Liu, et al. 2017; Yu et al. 2015). GRg1 significantly inhibited anaplastic thyroid carcinoma growth, and the survival time of mice with tumors was extended (Chang et al. 2016). By interfering with the activity of Haspin kinase and H3T3ph, GRg1 has been found to decrease the amount of Aurora B at the centromere. As a result, it can inhibit the growth and multiplication of cancer cells by impeding the critical mitotic processes, such as chromosome alignment and spindle dynamics, usually regulated by Aurora B. The depletion of Aurora B from the centromere is a crucial mechanism by which GRg1 suppresses cancer cell proliferation (Hong et al. 2022). Studies have indicated that GRg1 may serve as an effective adjuvant agent for enhancing the anti‐cancer functions of granulocytes that are inhibited by noradrenaline. The findings suggest that GRg1 could be a valuable addition to conventional cancer treatments, as it may help mitigate the inhibitory effects of noradrenaline on granulocyte function and enhance the effectiveness of anti‐cancer therapies (Zhu et al. 2023).

### 4.8 Comparative Perspective With Other Ginsenosides and Phytochemicals

GRg1 shares many biological properties with other major ginsenosides such as Rb1, Rg3, and Rh2, but each compound exhibits distinct pharmacological strengths and molecular targets. Rb1 is particularly recognized for its robust neuroprotective and anti‐apoptotic effects under oxidative and ischemic conditions, often linked to the activation of PI3K/Akt and inhibition of caspase‐mediated cell death (Yu et al. 2025). Rg3 and Rh2 are more potent in regulating tumor proliferation and immune responses, attributed to their ability to suppress NF‐κB activation and promote mitochondrial apoptosis in cancer cells (Xia et al. 2017). In contrast, Rg1 displays superior activity in enhancing synaptic plasticity, neurogenesis, and cognitive recovery, primarily through modulation of the Wnt/β‐catenin, PPARγ/HO‐1, and MAPK signaling pathways (Yang et al. 2023). When compared with other plant‐derived bioactives commonly used in complementary medicine, such as curcumin, resveratrol, and quercetin, Rg1 demonstrates a comparable antioxidant and anti‐inflammatory profile but exerts a more pronounced influence on neuronal differentiation and mitochondrial protection (Han et al. 2024). These observations suggest that Rg1 occupies a distinctive position among natural therapeutic agents, combining the cellular resilience typical of polyphenols with neuroregenerative and metabolic benefits more specific to ginsenosides.

## 5 Side Effects and Safety Data

**FIGURE 4:**
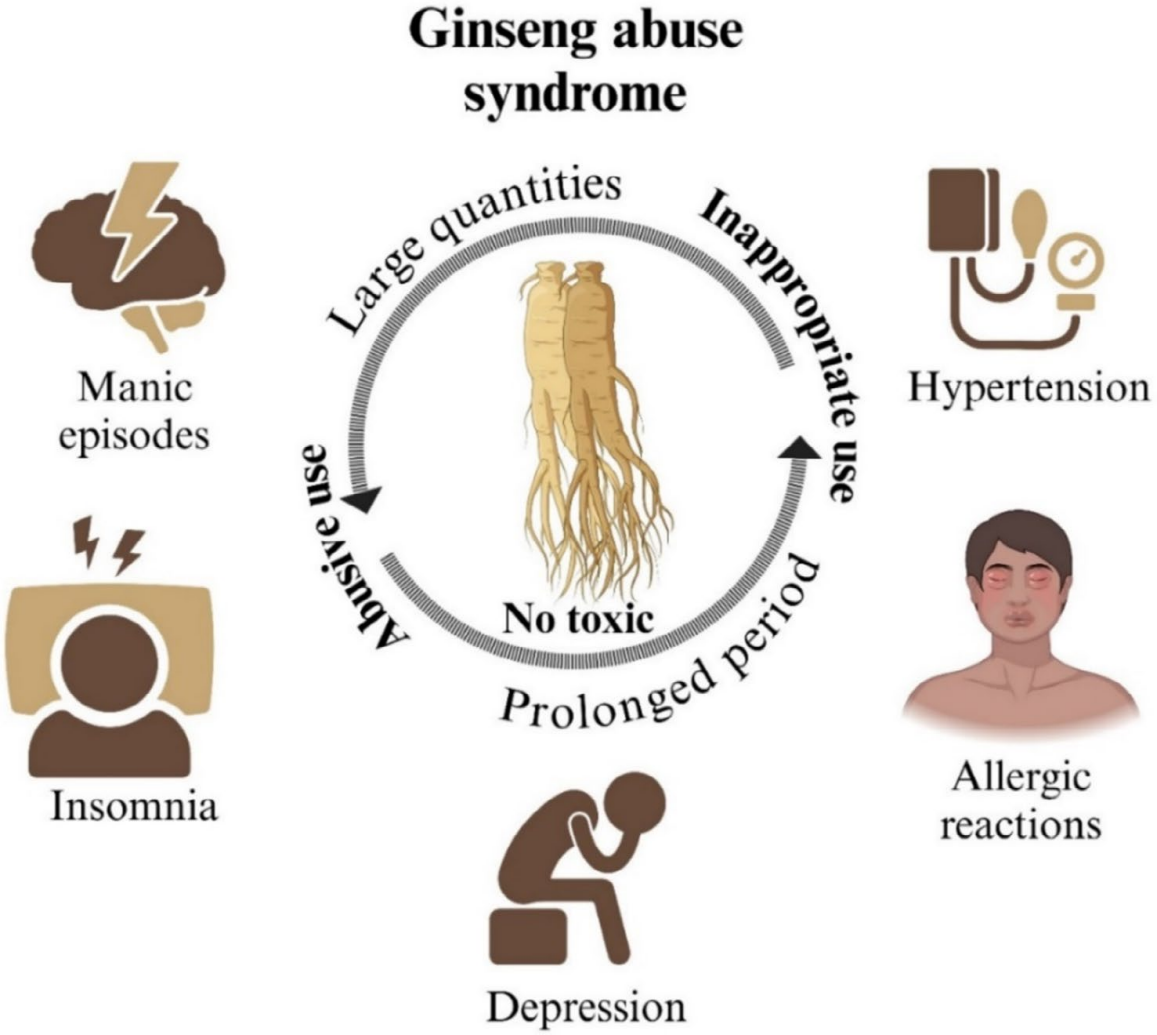
Adverse effects of ginseng abuse syndrome include manic episodes, insomnia, depression, hypertension, and allergic reactions.

GRg1 is one of the crucial pharmacological active components of ginseng. Despite limited studies on its safety and toxicity, results in GRg1 treatment of neurological and inflammatory diseases highlight its low toxicity and few side effects (Gao et al. 2020; Xie et al. 2015; Zhang et al. 2014) (Figure 4 and Table 2). On the other hand, ginseng has been in traditional medicine since ancient times, demonstrating significant benefits with minimal adverse effects (Jin et al. 2020; Ratan et al. 2021). Research on living organisms has shown that ginseng is well tolerated, even in high concentrations. Both short and long‐term studies in male and female F344/N rats and B6C3F1 mice revealed that a ginseng alcohol extract administered at doses up to 5 g/kg had no toxic or genotoxic effects, even with treatments lasting up to 2 years (Chan et al. 2011), similar results were obtained for Korean red ginseng extract G1899 in juvenile Sprague–Dawley rats (Kim et al. 2024). However, some clinical cases have shown that the abusive and inappropriate use of this product, also known as ginseng abuse syndrome, can produce side effects such as manic episodes, insomnia, nervousness, depression, allergic reactions, gynecomastia, cardiovascular and renal toxicity, and hypertension (Ernst 2002; Kiefer and Pantuso 2003; Kitts and Hu 2000). These reports are rare and mainly occur due to the misuse of ginseng, particularly its administration in large quantities or use for a prolonged period (Paik and Lee 2015). In the right proportions, ginseng can activate the immune system; however, excessive consumption may cause ginseng abuse syndrome by overstimulation of the immune system, causing symptoms similar to inflammation. A study by Deng et al. (2023) found that this overstimulation of inflammatory responses due to inappropriate use of ginseng occurs through the activation of the NG‐kB pathway; the research on this subject is still ongoing. Ginseng contains various active components that can interact negatively with different medications. One of these medications is warfarin, a widely used anticoagulant with a narrow therapeutic window, with an international normalized ratio of 2–3; warfarin commonly interacts with other active agents and should be treated carefully to avoid the risk of thrombosis. A randomized, double‐blind, and placebo‐controlled trial with a duration of 4 weeks and involving a group of 20 healthy subjects showed a significant decrease in the amount of warfarin in plasma in patients treated with ginseng compared with patients who received a placebo. In this trial, the patients were given warfarin for 3 days during weeks 1 and 4, and from the second week onwards, the patients were split into two groups: one group administered ginseng and the other a placebo. At the end of the 4 weeks, warfarin in plasma was determined (Yuan et al. 2004). Additionally, an in vivo study in rats showed a dose‐ and time‐dependent antagonism between ginseng and warfarin. This interaction led to a decrease in the concentration and activity of the anticoagulant, which becomes evident after a week and increases over time (Dong, Ma, et al. 2017). Also, other in vivo studies have presented that there is an interaction between ginseng and aspirin, finding that co‐administration of ginseng and aspirin led to an increase in the plasma concentration of aspirin and salicylates due to increased intestinal permeability (Tian et al. 2018).

**TABLE 2:** Summary of side effects, safety, and toxicity data of GRg1 and Ginseng extracts.

Type of study/model	Compound/formulation/dose/duration	Findings on safety and toxicity	Adverse or interaction effects	References
**In vitro studies**				
In vitro (mechanistic, immune activation)	Ginseng extract—high concentration exposure	Overactivation of immune and inflammatory responses.	↑ NF‐κB signaling → ↑ inflammation (mechanism of Ginseng abuse).	Deng et al. (2023)
**In vivo studies**				
In vivo (rodent models)	GRg1—variable therapeutic doses	Low toxicity; no behavioral or histopathological alterations; well tolerated.	None reported.	Gao et al. (2020), Xie et al. (2015) and Zhang et al. (2014)
In vivo (rats and mice, chronic exposure)	Ginseng alcohol extract—up to 5 g/kg for 2 years	No toxic or genotoxic effects; normal organ morphology and function.	None reported.	Chan et al. (2011)
In vivo (juvenile Sprague–Dawley rats)	Korean Red Ginseng Extract (G1899)—standardized dose	No histological abnormalities; normal biochemical parameters.	None reported.	Kim et al. (2024)
In vivo (rat)	Ginseng + warfarin—dose‐ and time‐dependent exposure	↓ Warfarin concentration and anticoagulant activity over time.	↑ Risk of clotting; ↓ bleeding control.	Dong, Ma, et al. (2017)
In vivo (rat)	Ginseng + aspirin—co‐administration	↑ Plasma aspirin and salicylate levels due to ↑ intestinal permeability.	↑ GI irritation; ↑ bleeding risk.	Tian et al. (2018)
**Human studies**				
Clinical trial (*n* = 20, healthy subjects)	Ginseng + Warfarin—4 weeks (warfarin administered weeks 1 & 4)	↓ Plasma warfarin levels; ↓ anticoagulant activity.	↑ Thrombosis risk if unmonitored.	Yuan et al. (2004)
Clinical case reports	Ginseng abuse (various formulations)—high‐dose or prolonged use	Adverse effects linked to overuse and chronic exposure.	↑ Manic episodes; ↑ insomnia; ↑ nervousness; ↑ depression; ↑ gynecomastia; ↑ hypertension; ↑ renal/cardiac toxicity.	Ernst (2002); Kiefer and Pantuso (2003), Kitts and Hu (2000) and Paik and Lee (2015)
Clinical observation	Ginseng + Insulin—concurrent administration	↓ Blood glucose beyond therapeutic range.	↑ Hypoglycemia risk; dose adjustment required.	Li and Gong (2015)
Pharmacological interaction (review)	Ginseng + Antidepressants (e.g., selegiline)—concurrent use	↓ Antidepressant bioavailability.	↑ Adverse CNS reactions.	Yang et al. (2020)
Pharmacological interaction (review)	Ginseng + immunosuppressants/Stimulants—concurrent use	↓ Immunosuppressant efficacy; synergistic effect with caffeine.	↑ Immune activation; ↑ tachycardia; ↑ blood pressure.	Choi and Song (2019)
Preclinical and clinical overview	Ginseng and GRg1—general assessment	Demonstrated overall safety and good tolerance at therapeutic doses.	Adverse reactions rare and dose‐dependent.	Jin et al. (2020) and Ratan et al. (2021)

Abbreviations: ↑, increase; ↓, decrease; AD, Alzheimer's disease; BBB, blood–brain barrier; BP, blood pressure; CNS, central nervous system; FSH, follicle‐stimulating hormone; GI, gastrointestinal; GRg1, Ginsenoside Rg1; HDL, high‐density lipoprotein; IL‐1β, interleukin‐1 beta; LH, luteinizing hormone; MAPK, mitogen‐activated protein kinase; NF‐κB, nuclear factor kappa B; NO, nitric oxide; PPARγ, peroxisome proliferator‐activated receptor gamma; TNF‐α, tumor necrosis factor‐alpha.

Ginseng has shown the ability to lower blood glucose, so taking it with insulin could cause an excessive decrease in glucose. If they are administered together, careful monitoring is required to adjust the dosage to prevent the risk of hypoglycemia (Li and Gong 2015). Other research has also suggested that ginseng decreases the bioavailability of antidepressant drugs such as selegiline, increasing the risk of adverse effects (Yang et al. 2020). Furthermore, due to its ability to activate the immune system, ginseng decreases the activity of immunosuppressive drugs. Finally, consuming ginseng with caffeine or stimulant drugs can cause tachycardia and an increase in blood pressure (Choi and Song 2019). Pharmacokinetic characteristics provide additional insight into Rg1's safety. Its limited oral bioavailability and extensive first‐pass metabolism result in low systemic exposure, reducing the likelihood of reaching toxic levels under normal therapeutic conditions. Moreover, although Rg1 undergoes hepatic metabolism, existing evidence indicates hepatoprotective rather than hepatotoxic effects, supporting its favorable safety profile even with prolonged use. Nevertheless, differences in formulation and absorption dynamics should be further investigated to ensure consistent exposure and predictable outcomes. While ginseng and its ginsenosides are generally safe, excessive or unregulated consumption especially from multiple supplement sources may still pose health risks, emphasizing the need for standardized dosing and careful product labeling.

## 6 Clinical Applications of GRg1

**FIGURE 5:**
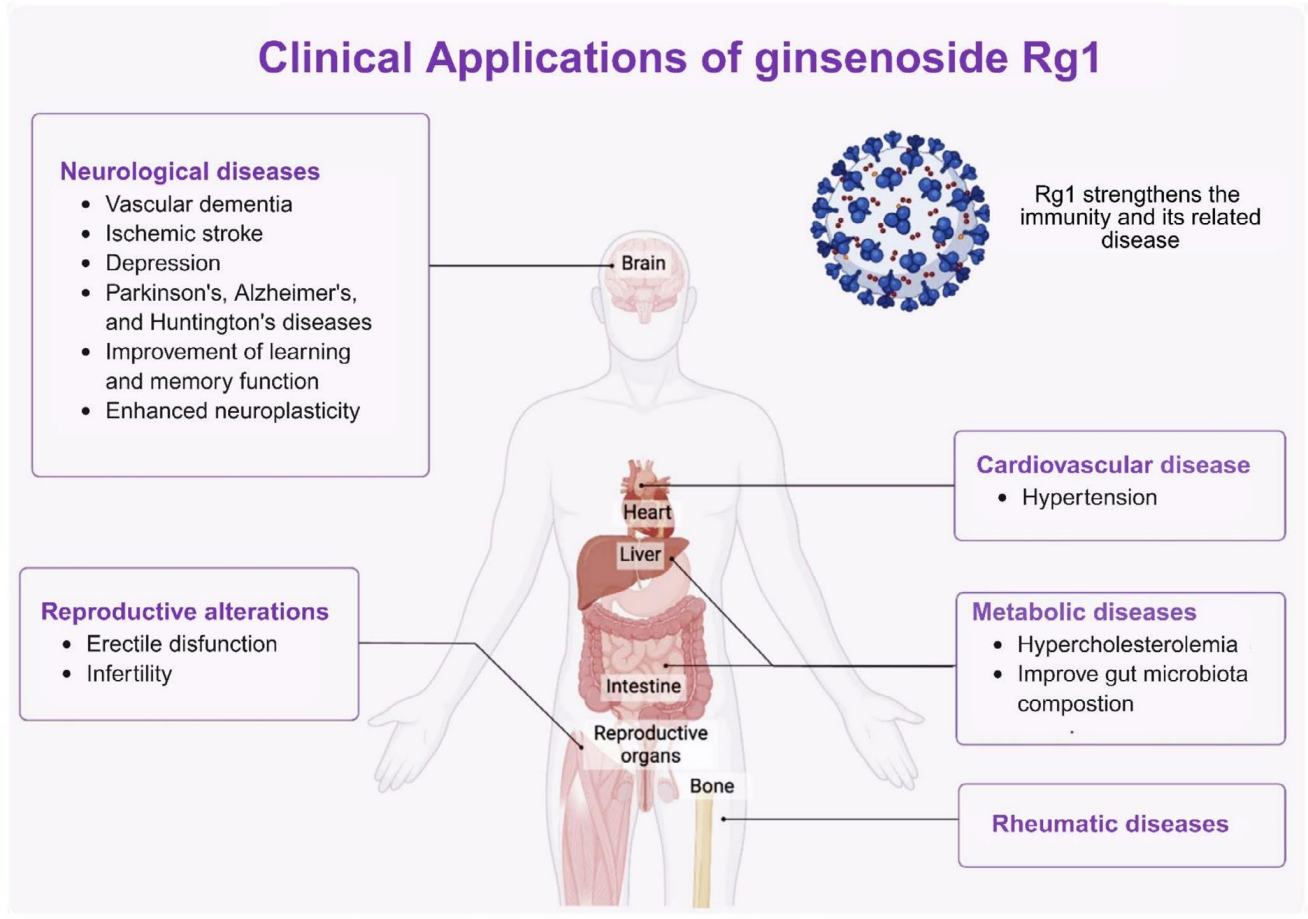
Overview of GRg1's neuroprotective and health benefits, including its roles in Parkinson's and Alzheimer's diseases, ischemic stroke, and improvements in gut microbiota.

Within the framework of complementary medicine, GRg1 holds significant potential as an adjuvant therapeutic compound. Its anti‐inflammatory, antioxidant, and neuroprotective properties suggest that Rg1 could complement conventional pharmacological treatments by reducing drug‐induced side effects, improving overall therapeutic outcomes, and enhancing patients' quality of life. For instance, its neuroprotective and cardioprotective actions may support recovery and reduce complications when used alongside standard therapies for neurodegenerative or cardiovascular diseases. However, standardized formulations, dosage optimization, and rigorous clinical validation are still required to ensure reproducibility and safety within integrative medical practice. Some scientific studies currently highlight and consider the multiple beneficial effects of GRg1 in clinical applications (Figure 5 and Table 3). We searched https://clinicaltrials.gov/ to find out the current use of GRg1. We found only seven studies that cover various research areas and analyze its therapeutic potential in treating dementia (NCT01761227 clinical trial), hypertension and hypercholesterolemia (NCT04069715 clinical trial), rheumatic disease (NCT03983408 clinical trial), ischemic stroke (NCT04142151, NCT02975076 clinical trials) and treatments related to reproductive alterations as erectile dysfunction and infertility (NCT02413099 and NCT02204826, respectively). Published clinical studies remain limited, but preliminary findings provide valuable insight. Most trials are early‐phase (Phase I or II), randomized, double‐blind, and placebo‐controlled, with small to moderate sample sizes (ranging from 30 to 240 participants). In a double‐blind, randomized, parallel placebo‐controlled trial carried out by, 240 patients with mild to moderate vascular dementia were administered three Fu Fang Dan Shen tablets three times per day for 24 weeks. The tablets contain tanshinone IIA 0.67 mg, salvianolic acid B 8.2 mg, *Panax* notoginsenosides R1 0.53 mg, ginsenoside Rb1 3.03 mg, and GRg1 2.73 mg. This is the first study that assesses the efficacy and safety of Fu Fang Dan Shen tablets for treating cognitive symptoms in patients with vascular dementia. The trial showed statistically significant cognitive improvements (*p* < 0.05); however, the results are limited by polyherbal formulation and lack of isolated GRg1 assessment. To date, no results have been posted on ClinicalTrials.gov. The use of GRg1 as a therapeutic method for the treatment of various neurological diseases, including PD, AD, and HD, stroke, cerebral infarction, ischemia–reperfusion injury, depression, improvement of learning and memory function, and stress is evaluated widely in preclinical studies (Sun et al. 2022). Specifically, the effect of GRg1 on depression has been evaluated recently. Wang et al. (2023) propose a new perspective for the development of a therapeutic strategy for depression, in which the authors demonstrated that GRg1 synergized with voluntary running exercise exerts greater efficiency in monitoring neuroinflammation, positively regulating the expression of neuronal trophic factors and synapse‐related proteins, thus improving neuronal structural damage, suggesting greater efficacy in the treatment of depression through anti‐inflammation and enhanced neuroplasticity (Wang et al. 2023). Given this evidence about the effectiveness of GRg1 in improving neurological diseases in preclinical models, it is necessary to conduct and explore clinical studies to gain insight into the mechanisms of action for treating these diseases in humans. Despite strong preclinical evidence, more controlled human studies are needed to confirm these effects in clinical populations. The efficacy of GRg1 in combination with other ginsenosides, such as Rb1, Rb2, Rd., Re, and notoginsenoside (Farlong Ginseng Plus *Panax notoginseng* extract), to decrease LDL cholesterol and blood pressure was evaluated in a randomized, placebo‐controlled, double‐blind, parallel study in human participants. The participants took two capsules daily in the morning, 30 min before a meal, for 12 weeks. According to the results, the Farlong NotoGinseng is promising as a compound to decrease LDL cholesterol and blood pressure compared to the placebo population (NCT04069715 clinical trial). GRg1 is utilized in neurodegenerative and hyperlipidemic diseases. It has also been tested to improve fatigue in patients with rheumatic disease in a randomized, double‐blind, placebo‐controlled trial study. Participants were randomly assigned to the Korean red ginseng or placebo group for 12 weeks and were instructed to consume two tablets of Korean red ginseng twice daily for 24 weeks. The results have not been posted (NCT03983408, clinical trial). No results have yet been posted (NCT03983408), limiting clinical interpretation at this stage.

**TABLE 3:** Summary of completed and ongoing clinical trials involving GRg1 and related ginseng formulations.

Trial ID	Condition/disease	Design and phase	Sample size	Intervention/formulation	Primary endpoints	Main outcomes (results/statistics)	Limitations/quality assessment	Status/source
NCT01761227	Vascular dementia	Randomized, double‐blind, placebo‐controlled, Phase II	240	Fu Fang Dan Shen tablets (2.73 mg GRg1 + Rb1, R1, tanshinone IIA)	Cognitive function (MMSE)	MMSE scores improved significantly vs. placebo (*p* < 0.05); cognitive subdomains also improved	Well‐designed RCT; polyherbal composition confounds isolated GRg1 effect.	Published/ClinicalTrials.gov
NCT04069715	Hypertension and hypercholesterolemia	Randomized, double‐blind, placebo‐controlled, parallel‐group Phase II	95	Farlong NotoGinseng (Ginseng Plus)—Panax notoginseng extract containing GRg1 and other ginsenosides	Change in serum LDL‐C (primary); blood pressure (secondary)	Study completed; no results posted or published as of Nov 2025	Robust protocol; lack of public data precludes efficacy assessment; multi‐component extract.	Completed/results not posted/ClinicalTrials.gov
NCT03983408	Rheumatic disease (Fatigue)	Randomized, double‐blind, placebo‐controlled, Phase II	120	Korean Red Ginseng (containing GRg1~1%–2%)	Fatigue Severity Scale (FSS)	Study completed; no posted results as of Nov 2025.	Adequate design; data not available; no peer‐reviewed publication.	Completed/no results posted/ClinicalTrials.gov
NCT04142151/NCT02975076	Ischemic stroke	Randomized, placebo‐controlled Phase II	60/120	GRg1‐related neuroprotective formulations (combination extracts)	Neurological recovery (NIHSS, mRS, infarct volume)	Ongoing; no interim data available.	Focused on GRg1's neuroprotective potential; results pending.	Ongoing / ClinicalTrials.gov
NCT02413099	Erectile dysfunction	Randomized, double‐blind, placebo‐controlled, Phase II	86 (19–40 years)	GCSB‐5 herbal formula (containing GRg1, Rb1, Rg3)	International Index of Erectile Function (IIEF‐5)	IIEF‐5 score significantly improved vs. placebo (*p* = 0.028); safe, no serious adverse events.	High methodological quality; short duration (8 weeks); polyherbal formulation limits specific GRg1 inference.	Published/ClinicalTrials.gov
NCT02204826	Male infertility (varicocele‐associated)	Randomized, double‐blind, placebo‐controlled, Phase II	80 (25–45 years)	Korean Red Ginseng (1.5 g/day for 12 weeks; contains GRg1, Rb1, Re)	Sperm concentration, motility, morphology, hormone levels (FSH, LH, testosterone)	Significant improvement in sperm parameters (*p* < 0.05); no hormonal change; minimal adverse events.	Moderate quality; good sample size; multi‐component extract precludes isolated GRg1 effect.	Published/ClinicalTrials.gov
NCT02202382	Male infertility	Randomized, double‐blind, placebo‐controlled, Phase II	80	Korean Red Ginseng (exact dose not disclosed)	Semen parameters and hormonal profile	Registered trial; no public results or linked publication.	Unknown completion status; data not available.	Completed/no results/ClinicalTrials.gov

Abbreviations: FSH, follicle‐stimulating hormone; FSS, Fatigue Severity Scale; GRg1, ginsenoside Rg1; IIEF‐5, International Index of Erectile Function–5; LDL‐C, low‐density lipoprotein cholesterol; LH, luteinizing hormone; MMSE, Mini‐Mental State Examination; mRS, modified Rankin Scale; NCT, ClinicalTrials.gov trial identifier; NIHSS, National Institutes of Health Stroke Scale; *p*, *p*‐value; R1, ginsenoside R1; Rb1, ginsenoside Rb1; RCT, randomized controlled trial; Re, ginsenoside Re; Rg3, ginsenoside Rg3.

The reproductive field is the most explored area for evaluating the efficacy of GRg1. In a randomized, double‐blind, placebo‐controlled clinical trial, the efficacy and safety of an herbal formula containing Rg1 and Rg2 ginsenosides were tested in the treatment of erectile dysfunction. Capsules were taken daily for 8 weeks in men aged 19–40 with erectile dysfunction, defined as the inability to maintain an erection sufficient for sexual performance. Participants had an International Index of Erectile Function score of ≤ 25. They were in stable, monogamous relationships, having unsuccessful erections more than 50% of at least four sexual attempts in a period (NCT02413099, clinical trial). Another randomized, placebo‐controlled, double‐blind clinical study assessed the effects of taking three capsules daily of Korean red ginseng, which contains GRg1, on semen parameters (sperm concentration, viability, percent motility, follicle‐stimulating hormone (FSH), testosterone, luteinizing hormone (LH) serum concentrations) in men of 25–45 years of age with infertility for at least 12 months. The Korean red ginseng was produced from the roots of 6‐year‐old red ginseng harvested in the Republic of Korea (NCT02204826, clinical trial). As of now, none of these reproductive studies have published their outcomes, and the individual contribution of GRg1 remains to be clarified.

Some areas could benefit from testing in humans, mainly because the results in preclinical trials have been promising, such as treating diabetes. Peng et al. (2022) assessed the effect of GRg1 on the improvement of gut microbiota in type 2 diabetes (DMT2). Based on a 4‐week treatment in which GRg1 was used as a supplement, an improvement in gut microbial composition was observed, resulting in a reduction in blood glucose levels and an improvement in insulin resistance, oxidative stress, inflammation, and lipid profile in rats with DMT2 (Peng et al. 2022). Zong et al. (2023) recently explored the mechanism of GRg1 in inflammation and β‐cell autophagy in type 1 diabetes (DMT1) and its therapeutic potential using a DMT1 mouse model injected with streptozotocin. At the end of the study, the authors concluded that there was an improvement in inflammation, body weight autophagy disorders, histological damage in the pancreas, and blood glucose (Zong et al. 2023). The administration of GRg1 also improved neuroinflammation in a rat model subjected to 5 min of asphyxia to explore the mechanisms by which underlying GRg1 in cognitive alterations. GRg1 can suppress neuroinflammation by mitigating glial cell activation and overexpression of proinflammatory cytokines (Wu et al. 2023). Another field that has yet to be evaluated in humans is the effect of GRg1 on microbiota. Also, this compound offers an option to treat immunomodulatory diseases and improve gut microbiota (Yousuf et al. 2022). It may be fundamental to evaluate the effectiveness of GRg1 in clinical settings for treating various diseases associated with increased inflammation, such as obesity, DMT2, colitis, and metabolic syndrome. This could also encompass potential impacts on immune organs, including their association with disease severity and/or immune response dysfunction in patients with COVID‐19 (Yousuf et al. 2022). Despite promising pharmacological results, the clinical translation of GRg1 remains limited by its poor oral bioavailability and short half‐life. To address these challenges, novel formulation strategies have been proposed. Nanoparticle‐ and liposome‐based delivery systems, solid lipid nanoparticles, and polymeric micelles have shown potential to improve intestinal absorption, protect Rg1 from enzymatic degradation, and prolong systemic circulation (Kim et al. 2018; Yu et al. 2025). Additionally, nanoemulsions and hydrogel‐based carriers may facilitate targeted delivery, particularly to the brain, enhancing Rg1's therapeutic performance in neurodegenerative and cardiovascular conditions (H. Wang et al. 2021). Future clinical trials should therefore integrate such optimized formulations to ensure improved pharmacokinetics and reproducibility of therapeutic outcomes.

## 7 Limitations and Research Gaps

Although extensive studies have demonstrated the pharmacological potential of GRg1, several limitations continue to restrict its broader clinical application. Issues such as low bioavailability, limited clinical evidence, possible drug interactions, and the absence of standardized extracts remain major challenges that need to be addressed through further pharmacokinetic and clinical research (Gao et al. 2020). Variability among different ginseng species also complicates research, as GRg1 content and biological activity can differ depending on the Panax species, cultivation conditions, and extraction methods. Current data on these interspecies variations are limited, highlighting the need for comparative studies to better understand and safely harness GRg1's therapeutic potential. One of the main barriers to the effective use of GRg1 is its poor oral absorption and rapid metabolism, which significantly reduce its bioavailability. This limitation underscores the importance of developing alternative delivery systems or formulations that enable controlled, localized, and sustained release. This is particularly relevant for its neuroprotective applications, since GRg1 shows minimal penetration across the blood–brain barrier, resulting in limited central nervous system activity (Kim et al. 2021; Zhai et al. 2021). Further research is required to clarify the action mechanisms of GRg1 and the pathways it activates to carry out its hepatoprotective, cardioprotective, anti‐aging, and anti‐inflammatory activity (Guan et al. 2023). As well as information about GRg1 absorption, distribution, metabolism, and excretion profiles. This knowledge would help to achieve the optimal doses for effective action. Although there are several studies on the behavior of GRg1 in vitro and preclinical studies in animals, clinical research in humans is still a topic to explore, with only a few studies on specific diseases being found (see Section 6—Clinical applications of GRg1). While these studies are a good starting point, larger sample sizes are needed to corroborate the results and demonstrate the effectiveness of GRg1 for the treatment of these diseases. Also, more information about the safety and short‐, medium‐, and long‐term side effects is required, as well as the appropriate doses of use and the treatment duration to ensure safety, and information on acute, chronic, and teratogenic toxicity. Ginseng has been shown to interact with some drugs, and it may be dangerous to use it in combination with other treatments (see Section 5. Side effects and safety data). GRg1 is one of the main active ingredients of ginseng that could be responsible for these interactions. Therefore, research about the co‐administration of GRg1 with different pharmaceutical treatments is crucial, especially for diseases in which its effectiveness has been demonstrated. Since its synergistic effects could be hazardous, so this information would be helpful for the safety and proper use of this active agent. GRg1 is extracted from ginseng, which is a natural source. Due to natural product compositions varying for environmental conditions, GRg1 extracts can change depending on the source, representing a limitation for research (Kim et al. 2010; Mohanan et al. 2018). Establishing strict quality control and standardization protocols will help ensure consistency across studies and clinical applications. Addressing these limitations through integrated in vitro, in vivo, and clinical research using standardized GRg1 formulations will provide a stronger foundation for its safe and effective use both as a therapeutic agent and as a complementary supplement to enhance overall health and quality of life.

## 8 Conclusions and Future Outlooks

Ginseng has long been valued in traditional Chinese medicine for its wide‐ranging therapeutic properties, including regulation of blood pressure, immune modulation, and metabolic balance. Among its active components, GRg1 has emerged as a key bioactive molecule with demonstrated neuroprotective, cardioprotective, anti‐inflammatory, and anti‐aging effects. These findings highlight GRg1's potential not only as a pharmacologically active compound but also as a promising agent within the framework of complementary medicine. In this context, GRg1 may serve as an effective adjuvant therapy to complement conventional treatments—helping to mitigate drug‐induced side effects, enhance therapeutic efficacy, and improve patients’ quality of life. However, despite encouraging preclinical and limited clinical evidence, further rigorous, large‐scale clinical trials are required to establish its efficacy, safety, and optimal dosing. Future research should also focus on standardization, mechanistic clarification, and regulatory integration to facilitate the safe incorporation of GRg1 into evidence‐based complementary and integrative medicine.

## Author Contributions

**Zainab M. Almarhoon:** investigation, writing – original draft, writing – review and editing, data curation, methodology, visualization. **Sheila I. Peña‐Corona:** data curation, writing – review and editing, writing – original draft, visualization, methodology, investigation. **Hernán Cortés:** writing – original draft, writing – review and editing, visualization, investigation, methodology, data curation. **William N. Setzer:** data curation, writing – review and editing, writing – original draft, investigation, methodology, validation, visualization. **Enrique Lima:** data curation, methodology, visualization, writing – review and editing, writing – original draft, investigation. **Gerardo Leyva‐Gómez:** investigation, methodology, validation, visualization, writing – review and editing, writing – original draft, supervision, data curation. **Jen‐Tsung Chen:** writing – review and editing, writing – original draft, investigation, methodology, data curation. **Javad Sharifi‐Rad:** investigation, conceptualization, writing – original draft, writing – review and editing, visualization, validation, methodology, project administration, data curation, supervision. **Daniela Calina:** investigation, writing – original draft, writing – review and editing, validation, visualization, methodology, data curation, project administration. **Lorena Duarte‐Peña:** investigation, writing – original draft, writing – review and editing. **Rajesh Kaverikana:** data curation, methodology, visualization.data curation, writing – review and editing, writing – original draft, visualization, methodology, investigation. **Shivaprasad Shetty Mangalpady:** writing – original draft, writing – review and editing, visualization, investigation, methodology, data curation. **Vinayaka Babu Shet:** writing – original draft, writing – review and editing. **Nikshitha Manjeshwar:** visualization, investigation, methodology, data curation, investigation, writing – original draft, writing – review and editing, data curation, methodology, visualization.

## Funding

The authors have nothing to report.

## Conflicts of Interest

The authors declare no conflicts of interest.

## Data Availability

Data sharing not applicable to this article as no datasets were generated or analysed during the current study.
